# Temporal lobe disconnection in drug-resistant epilepsy associated with hippocampal sclerosis: how I do it

**DOI:** 10.1007/s00701-026-06826-2

**Published:** 2026-03-07

**Authors:** Antonio Leocata, Roberta di Giacomo, Vittoria Nazzi, Michele Rizzi

**Affiliations:** 1 Division of Neurosurgery, Department of Medical and Surgical Specialties, Radiological Sciences and Public Health, University of Brescia, Brescia, Italy; 2 Functional Neurosurgery Unit, Neurosurgery Department, Foundation IRCCS Carlo Besta Neurological Institute, Milan, Italy; 3 Clinical and Experimental Epileptology and Sleep Disorders Unit, Foundation IRCCS Carlo Besta Neurological Institute, Milan, Italy

**Keywords:** Temporal lobe epilepsy, Hippocampal sclerosis, Epilepsy surgery, Temporal lobe disconnection

## Abstract

**Background:**

Epilepsy surgery is the treatment of choice for drug-resistant epilepsy associated with hippocampal sclerosis. Patients over 50 years of age have an increased risk of postoperative complications after epilepsy surgery.

**Methods:**

The authors present an operative video demonstrating right temporal lobe disconnection in a case of hippocampal sclerosis, along with an anatomical introduction and a section providing strategies to minimize complications.

**Results:**

In selected cases, temporal lobe disconnection represents a viable alternative to resective procedures.

**Supplementary Information:**

The online version contains supplementary material available at 10.1007/s00701-026-06826-2.

## Introduction

Temporal lobe epilepsy (TLE) is the most prevalent form of epilepsy [5]. In patients with drug-resistant epilepsy who undergo surgery, hippocampal sclerosis represents the most frequent histopathological finding among adults, occurring in approximately 36.4% of cases [1]. In cases of drug-resistant TLE associated with hippocampal sclerosis, anterior mesial temporal lobectomy (AMTL) is considered the most effective surgical treatment, with 78.6% of patients achieving Engel Class I seizure outcomes at a mean follow-up of 11.6 years [4]. Patients over the age of 50 experience a 30% increased risk of significant adverse events, including cerebral infarctions and subdural or intracerebral hematomas [7].

## Relevant surgical anatomy

The Sylvian fissure, as well as the superior (T1), middle (T2) and inferior (T3) temporal gyri, must be exposed through the craniotomy. The vascular structures of the Sylvian fissure and temporal lobe—including the vein of Labbé and other venous anastomoses—are carefully identified and preserved. The temporal horn of the lateral ventricle serves as the primary anatomical landmark for dissection. The anterior choroidal point is a key landmark to perform a safe amigdalectomy. After uncus and amygdala resection, the free edge of the tentorium and the posterior cerebral artery become clearly visible, along with the cerebral peduncle and optic tract.

## Description of the technique

**Fig. 1:**
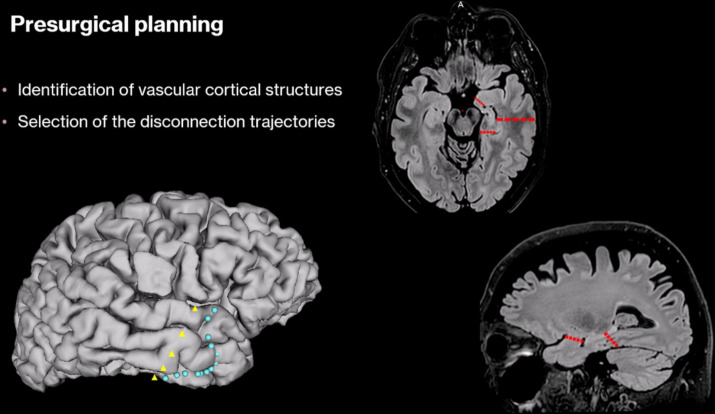
Presurgical 3D reconstruction and disconnection lines (red dashes). Yellow triangles: posterior limit of disconnection. Turquoise dots: atypical inferior anastomotic vein

The patient is placed in the supine position with the head fixed in a head holder and rotated 45° to the contralateral side. Neuronavigation is employed and the preoperatively reconstructed surgical scene is visualized on a dedicated screen (Fig. 1).

Necessary instrumentation includes bipolar forceps, the ultrasonic aspirator, and surgical curettes for subpial dissection.

**Fig. 2:**
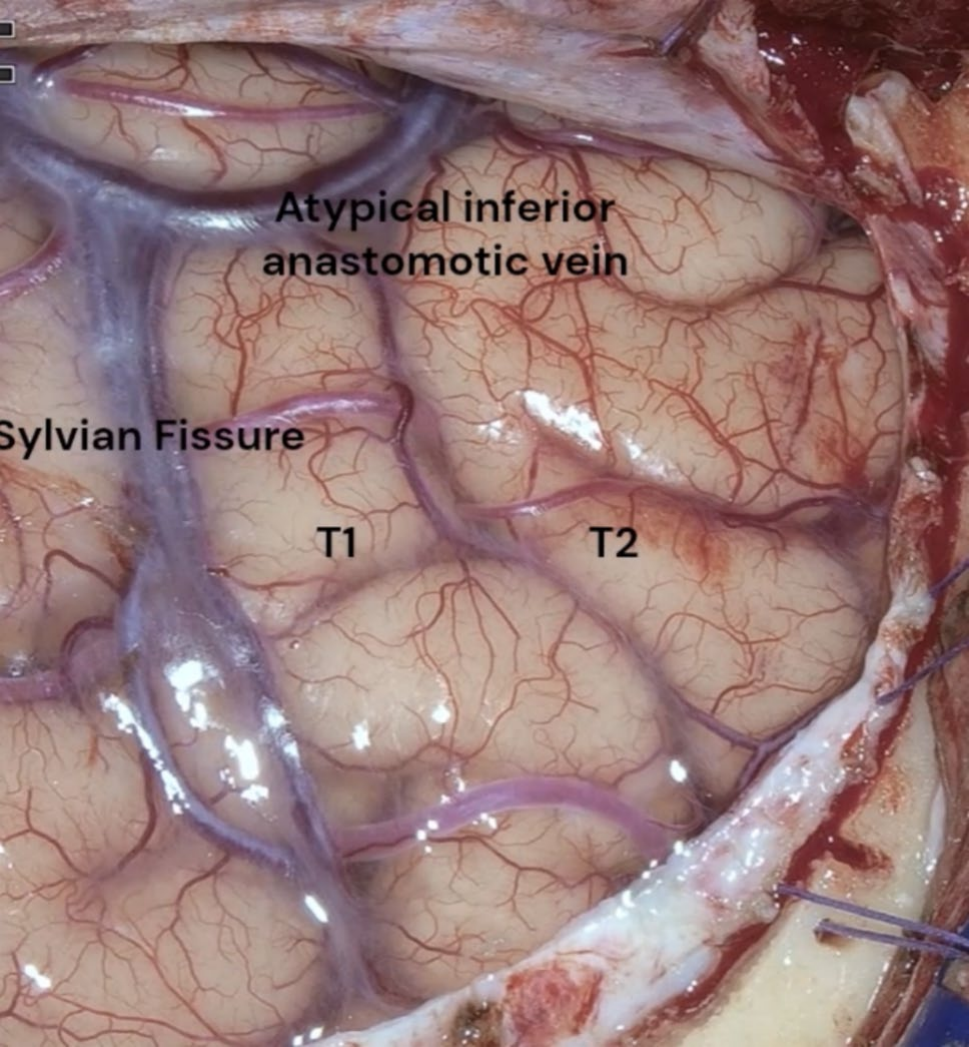
Cortical exposure after dura mater opening

A frontotemporal arciform incision is performed, and the myocutaneous flap is reflected to expose the zygomatic process of the temporal bone. The frontotemporal craniotomy allows visualization of the Sylvian fissure and the superior (T1) and a large middle (T2) temporal gyri (Fig. 2).

The first step consists of identifying the temporal horn of the lateral ventricle. Posterolateral disconnection is achieved by sectioning from T2 to T4. Subpial dissection is used to preserve the arachnoid and the vascular structures.

From the temporal horn of the lateral ventricle, the choroidal fissure is identified, and a microcottonoid is placed to protect the thalamus. Posteromedial disconnection is then carried out by sectioning fimbria, hippocampus and parahippocampal cortex.

Removal of the anterior portion of T1 reduces the need for retraction during the anteromesial disconnection phase; this step is performed by sectioning the uncus and amygdala, exposing the free edge of the tentorium and connecting the dissection plane with the temporal horn of the lateral ventricle.

The head of the hippocampus is resected for histopathological examination.

The final step involves completing the disconnection plane between T1 and T2.

Dural closure is generally performed by using dural substitutes.

### Indications

Temporal lobe disconnection has been proposed as an alternative to resective procedures, such as AMTL or selective amygdalo-hippocampectomy (SAH), for the treatment of drug-resistant temporal lobe epilepsy [2, 3]. In contrast to temporal lobe disconnection, different types of SAH, from the trans-sylvian to the trans-T2 (to cite the more frequent), try to spare the connection of the temporal pole (and other neocortical temporal region, on the base and more mesial) with the rest of the brain.

Patients older than 50 years present a 30% increased risk of postoperative complications. Temporal lobe disconnection—by preserving the lobe in situ—may reduce the risk of complications such as subdural collections (hematomas or hygroma), particularly in elderly patients or those with cerebral atrophy.

The proposed temporal lobe disconnection can be proposed also in the left-hand side, in every dominant side for language, as a temporal lobectomy is proposed. The limits of the disconnection are the same limits of resection with the difference of leaving in place the anterior portion of the temporal lobe. Consequently, the choice of a left temporal disconnection is based on the classical concept of anatomo-electro-clinical correlations.

### Limitations

Due to the narrow surgical corridors, this procedure requires a high level of surgical expertise. The results of temporal lobe disconnection on larger scale are by far not comparable to those of traditional resective methods and more extensive studies with longer follow-ups are needed. Temporal lobe disconnection is not indicated in cases of lesional temporal lobe epilepsy.

### How to avoid complications

1. Surgical planning by means of 3D brain models, the use of intraoperative neuronavigation system and identification of anatomical intraoperative landmarks are essential strategies to reduce the risk of incomplete disconnection.
2. Subpial dissection decreases the risk of injury to vascular structures.
3. The disconnection lines must not be too narrow in order to avoid difficult surgical maneuvers. The second operator is decisive in facilitating disconnections.
4. Tractography with intraoperative projection of the model on the microscope is an option to reach a reduction of the contralateral superior quadrant defect, as proposed for the temporal lobectomy [6].

### Specific information for the patient

Patients must be informed about the alternative to temporal lobe disconnection, in particular about the classical AMTL and SAH, or laser-based techniques (whereas available).

## Key points

1. Temporal lobe disconnection (TLD) is proposed as an alternative to anterior mesial temporal lobectomy for drug-resistant temporal lobe epilepsy with hippocampal sclerosis, particularly in older patients at increased risk of postoperative complications
2. The goal of TLD is to achieve functional disconnection of temporal lobe while preserving parenchyma and its vascularization
3. Comprehensive evaluation includes video-EEG with ictal recording, structural and functional MRI, PET, and neuropsychological testing. In inconclusive cases, invasive EEG monitoring with foramen ovale electrodes or SEEG is mandatory
4. 3D presurgical planning and intraoperative neuronavigation facilitate accurate identification of anatomical landmarks and minimize the risk of incomplete disconnection
5. Exposure of the Sylvian fissure, superior (T1) and middle (T2) temporal gyri, and identification of key vascular structures—particularly the vein of Labbé—are mandatory for safe disconnection
6. Subpial dissection and meticulous preservation of the arachnoid and vascular structures reduce the risk of vascular injury and postoperative hematoma
7. Temporal horn of the lateral ventricle, choroidal fissure and free tentorial edge serve as key landmarks
8. The hippocampal head must be resected for histopathological examination
9. Patients must be informed about possible postoperative complications. Contralateral superior quadrantanopia is the most frequent postoperative finding
10. Postoperative MRI allows evaluation of the disconnection planes
11. Antiseizure medications are gradually tapered following surgery.

## Supplementary Information

Below is the link to the electronic supplementary material.

ESM 1 Supplementary Material 1 (MP4 431 MB)

## Data Availability

No datasets were generated or analysed during the current study.

## Abbreviations

TLE: Temporal lobe epilepsy
AMTL: Anterior mesial temporal lobectomy
TLD: Temporal lobe disconnection
ASM: Anti Seizure Medication
A.Ch.A: Anterior Choroidal Artery
PCA: Posterior Cerebral Artery
